# ﻿*Panstrongylusnoireaui*, a remarkable new species of Triatominae (Hemiptera, Reduviidae) from Bolivia

**DOI:** 10.3897/zookeys.1104.81879

**Published:** 2022-06-14

**Authors:** Hélcio R. Gil-Santana, Tamara Chavez, Sebastián Pita, Francisco Panzera, Cleber Galvão

**Affiliations:** 1 Laboratório de Diptera, Instituto Oswaldo Cruz, Av. Brasil, 4365, 21040-360, Rio de Janeiro, RJ, Brazil; 2 Instituto Nacional de Laboratorios de Salud, Laboratorio de Entomología Médica, La Paz, Bolivia; 3 Universidad de la República, Facultad de Ciencias, Sección Genética Evolutiva, Montevideo, Uruguay; 4 Laboratório Nacional e Internacional de Referência em Taxonomia de Triatomíneos, Instituto Oswaldo Cruz, Fiocruz, Pavilhão Rocha Lima, Avenida Brasil, 4365, Manguinhos, RJ, Brazil

**Keywords:** Chagas disease vectors, genitalia, kissing bug, taxonomy

## Abstract

*Panstrongylusnoireaui***sp. nov.** from Bolivia is described based on male and female specimens. Although morphologically almost indistinguishable from *Panstrongylusrufotuberculatus* (Champion, 1899), the new species shows remarkable chromosome and molecular features, which are very distinctive among all others *Panstrongylus* species. The new species is also separated by some characteristics of the processes of the endosoma of the male genitalia. An updated key for species of *Panstrongylus* is provided.

## ﻿Introduction

The Triatominae are classified as a subfamily of Reduviidae (Hemiptera, Heteroptera), defined by their blood-sucking habit and morphological adaptations associated with host-finding and blood-feeding (Schofield and Galvão 2009). Currently, there are 154 extant and three fossil species distributed in 18 genera and five tribes in Triatominae (Dale et al. 2021; Galvão 2021). All of them are considered as potential vectors of the protozoan *Trypanosomacruzi* (Chagas, 1909), the causative agent of Chagas disease or American trypanosomiasis, which still is a serious health problem to most Latin American countries (Arias et al. 2021). Among the five tribes included in Triatominae, the tribe Triatomini has ten genera (Galvão 2021), among which *Panstrongylus* Berg, 1879 has several species involved in the transmission of *T.cruzi* in Central and South America (Patterson et al. 2009).

Berg (1879) created the genus *Panstrongylus* for a single new species he was describing, *P.guentheri* Berg, which is the type species of this genus by monotypy. He regarded *Panstrongylus* as being close to *Lamus* Stål, 1859. Champion (1899) described *Lamusrufotuberculatus* based on a single male from Panama. As *Lamus* Stål, 1859 was a preoccupied name by *Lamus* Stål, 1854, a genus of Pentatomidae, Kirkaldy (1904) proposed a new name for it, *Mestor* Kirkaldy. The species was transferred to *Panstrongylus* by Pinto (1931), with the resulting new combination, *P.rufotuberculatus* (Champion). Pinto (1931) argued that *Mestor* should be considered a synonym of *Panstrongylus*. Usinger (1939), however, maintained these two latter genera as valid. Lent and Pifano (1940) strongly defended the synonym of *Mestor* under *Panstrongylus* as proposed by Pinto (1931), which was accepted by subsequent authors so far, with the exception of Usinger (1944), who still considered the validity of both genera. The main novelties involving species of *Panstrongylus*, after the classical taxonomical treatment of the group by Lent and Wygodzinsky (1979), were recently summarized by Monteiro et al. (2018), Costa et al. (2021), Galvão (2021), and Paiva et al. (2022).

*Panstrongylus* has been considered monophyletic based on morphological features (Lent and Wygodzinsky 1979). However, molecular studies using several nuclear (ITS-2, 18S, 28S, ultraconserved elements), and mitochondrial (16S, coI, coII, cyt b) markers demonstrated a paraphyletic status for *Panstrongylus* (Hypša et al. 2002; Marcilla et al. 2002; Justi et al. 2014; Monteiro et al. 2018; Kieran et al. 2021; Pita et al. 2021).

*Panstrongylusrufotuberculatus* has been recorded in several countries: Mexico, Panama, Costa Rica, Colombia, Venezuela, French Guiana, Suriname, Ecuador, Peru, Bolivia, Brazil, and Argentina (Galvão et al. 2003; Bérenger et al. 2009; Patterson et al. 2009; Hiwat 2014).

Beginning with the observation of Lent and Pifano (1940), several authors have recorded natural infection of *P.rufotuberculatus* with *T.cruzi* as summarized by Patterson et al. (2009). These latter authors also summarized host observation to this species, which have included different wild and domestic mammals and humans as well.

*Panstrongylusrufotuberculatus* has been considered as a sylvatic species, which frequently invades human dwellings as it is attracted by electric light (Lent and Wygodzinsky 1979; Salomón et al. 1999; Patterson et al. 2009; Gorla and Noireau 2010). However, truly domestic populations were reported only in some areas of southern Ecuador (Abad-Franch et al. 2001), while breeding colonies have been found inside dwellings in Bolivia and Peru too (Marín et al. 2007; Gorla and Noireau 2010). This species has been incriminated as a vector of Chagas disease in Andean and coastal foci of Ecuador, whereas its presence in the municipality of Amalfi (Antioquia, Colombia) is considered as a major epidemiological risk factor (by being the second most common triatomine caught inside buildings) (Wolff et al. 2001; Patterson et al. 2009; Gorla and Noireau 2010).

Following the description of the male holotype by Champion (1899), Lent and Pifano (1940), Lent and Jurberg (1975) and Lent and Wygodzinsky (1979) provided thorough redescriptions of *P.rufotuberculatus*. These latter authors and some others, e.g., Salomón et al. (1999) and Hiwat (2014), have emphasized the morphological and chromatic variation of this species. Its male genitalia was thoroughly described and figured by Lent and Jurberg (1975), while Lent and Wygodzinsky (1979) commented on some features of it.

Although almost all species of *Panstrongylus* have been recorded as possessing only a paired [lateral] process in the endosoma (Lent and Jurberg 1975; Lent and Wygodzinsky 1979; Jurberg et al. 2001; Papa et al. 2003; Bérenger and Blanchet 2007; Ayala 2014, pers. comm.), *P.rufotuberculatus* has been recorded as the only species of the genus with two paired [lateral] process in the endosoma (Lent and Jurberg 1975; Lent and Wygodzinsky 1979). This latter feature, together with presence of body scalelike setae and an apically bilobed clypeus were all considered the three apomorphies of this species in the phylogenetic scheme proposed by Lent and Wygodzinsky (1979). These authors, however, recorded that these latter two features in *P.rufotuberculatus* were also present as what they considered as plesiomorphic states among *Panstrongylus* spp. (not scalelike setae and not bilobed [unilobed] clypeus, respectively). Salomón et al. (1999), by their turn, recorded scalelike setae but only unilobed clypeus among specimens of *P.rufotuberculatus* from Argentina.

The female genitalia in Triatominae was considered uniform by several authors and, by consequence, without taxonomic significance (Lent and Wygodzinsky 1979; Rodrigues et al. 2018). However, in just over the last decade several works, summarized by Rodrigues et al. (2018), have proven that the study of female genitalia is useful in many cases to the taxonomy in Triatominae, showing that in fact they present characteristics of diagnostic value. Rodrigues et al. (2018) studied, compared, and figured with SEM images the female genitalias of 26 species belonging to seven genera of Triatominae, including *P.rufotuberculatus*.

Pita et al. (2021) through a multidisciplinary approach suggested speciation within populations of *P.rufotuberculatus*. Extensive chromosomal analyses supported that the two chromosomal groups could represent different closely related species. Molecular and morphometric analyses reinforced the marked cytogenetic differences. Therefore, they proposed that Bolivian individuals constituted a new *Panstrongylus* species, which is herewith described as *Panstrongylusnoireaui* sp. nov., based on male and female specimens. A detailed morphological description of the new species is provided, comparing its characteristics with extensive previous data from the literature, and examination of specimens of *Panstrongylusrufotuberculatus* from different countries.

## ﻿Materials and methods

All type specimens of *Panstrongylusnoireaui* sp. nov. and non-type specimens of *Panstrongylusrufotuberculatus* examined here are deposited in the “Coleção de Triatomíneos do Instituto Oswaldo Cruz” (**CTIOC**) of the “Laboratório Nacional e Internacional de Referência em Taxonomia de Triatomíneos” (**LNIRTT**) at Oswaldo Cruz Institute, Rio de Janeiro, Brazil.

All the figures were produced by the first author (HRG-S). The photographs were obtained using digital cameras (Nikon D5600 with a Nikon Macro Lens 105 mm, Sony DSC-W830). Drawings were made using a camera lucida. Dissections of the male genitalia were made by first removing the pygophore from the abdomen with a pair of forceps and then clearing it in 20% NaOH solution for 24 hours. The dissected structures were studied and photographed in glycerol. Images were edited using Adobe Photoshop CS6.

General morphological terminology and particularly those of the male genitalia portions used here follows mostly Lent and Wygodzinsky (1979), while to female genitalia Rodrigues et al. (2018) is followed. However, some portions of head terminology follow Schuh and Weirauch (2020), in which “rostrum” is named labium. In this particular, the [visible] segments of the labium are numbered II to IV, given that the first segment is said to be lost or fused to the head capsule (Weirauch 2008). In addition, according to Schuh et al. (2009), for the convention of numbering labial segments in the Reduviidae, the apical segment should be considered as number four and then counted backwards towards the base. Jugum (pl. juga) *sensu*Lent and Wygodzinsky (1979) is named mandibular plate. In male genitalia, “vesica” as recognized by Lent and Jurberg (1975) and Lent and Wygodzinsky (1979) has been considered to be absent in reduviids. The assumed equivalent structure in reduviids is a somewhat sclerotized appendage of the phallosoma or the endosoma (Forero and Weirauch 2012), but not the homologous vesica that occurs in other heteropterans such as Pentatomomorpha (Rédei and Tsai 2011). Thus, this term is not used here for the median process of endosoma, which is named as such.

When describing label data, a slash (/) separates different labels.

## ﻿Results

### ﻿Taxonomy

**Triatominae Jeannel, 1919**

**Triatomini Jeannel, 1919**

#### *Panstrongylus* Berg, 1879

##### ﻿ Panstrongylus noireaui sp. nov.

Taxon classificationAnimaliaHemipteraReduviidae

8D475B70-8E83-5BD6-A717-FA312A9C6F7C

http://zoobank.org/5126A8F8-0FEA-4265-8669-41D03756693E

Figs 1 –11

###### Type material.

Bolivia, La Paz Department, Ildefonso de las Muñecas Province, Ayata locality, community of Camata (15°14'22"S, 68°44'52"W), 2004, ***Holotype***, male [13177, CTIOC]. ***Paratypes*** 2 male [13178, 13179, CTIOC], 2 female paratypes [13180, 13181, CTIOC].

**Figures 1–6.**
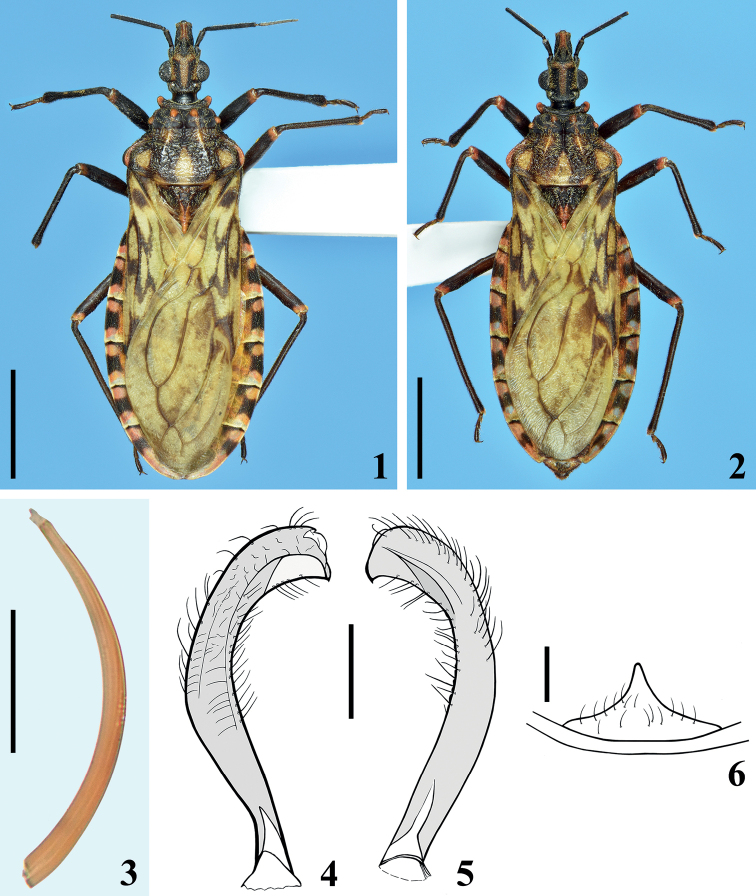
*Panstrongylusnoireaui* sp. nov. **1, 2** habitus, dorsal view **1** male holotype **2** female paratype **3** body seta **4, 5** left paramere **4** ventral view **5** dorsal view **6** median process of pygophore, dorsal view. Scale bars: 5.0 mm (**1, 2**); 0.5 mm (**4, 5**); 0.2 mm (**6**); 0.04 mm (**3**).

###### Diagnosis.

*Panstrongylusnoireaui* sp. nov. can be morphologically separated from *P.rufotuberculatus* mainly by the lateral processes of endosoma, which are smooth in the former and with numerous and delicate teeth at apical portion in the latter species. Additionally, whilst the elongate process which is present on the ventral portion of the lateral flap like prominences of the dorsal phallothecal plate is thinner and almost straight in *P.noireaui* sp. nov., it is curved and larger in *P.rufotuberculatus*.

###### Description.

**Figures 7–11.**
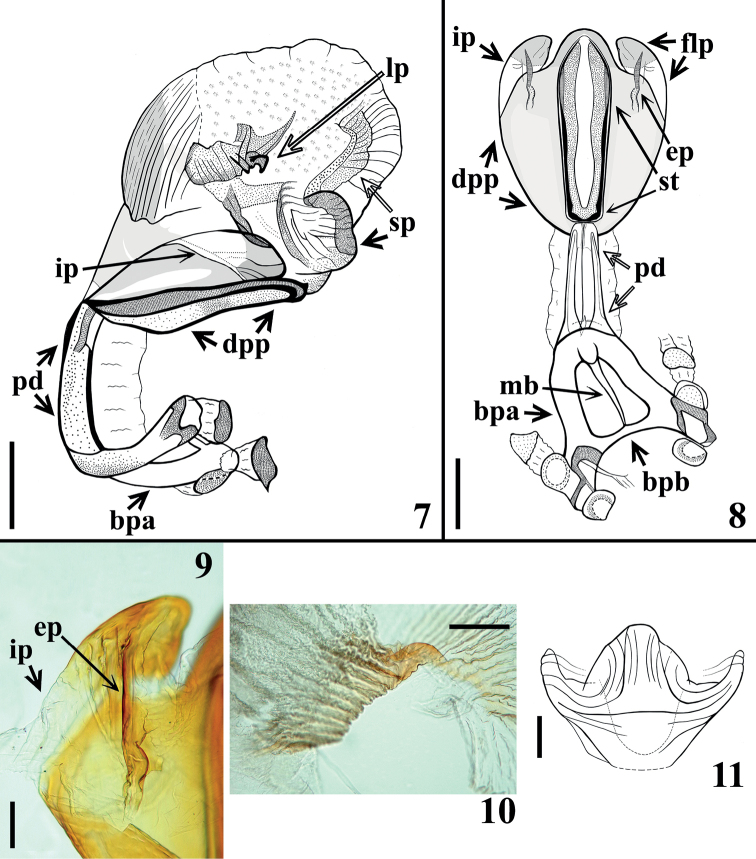
*Panstrongylusnoireaui* sp. nov., male genitalia. **7** phallus, lateral view **8** articulatory apparatus, dorsal phallothecal plate and struts, dorsal view **9** latero-apical portion of dorsal phallothecal plate (flap like prominence), ventral view **10** lateral process of endosoma, lateral view **11** median process of endosoma, posterior view. Abbreviations: **bpa** basal plate arm, **bpb** basal plate bridge, **dpp** dorsal phallothecal plate, **ep** elongate process, **flp** lateral flap like proeminence, **ip**, intermediate less sclerotized portion, **lp** lateral process of endosoma, **mb** median bridge, **pd** pedicel, **sp** subapical median process of endosoma, **st** struts. Scale bars: 0.5 mm (**7, 8**); 0.2 mm (**10**); 0.1 mm (**9, 11**).

**Male.** Figs 1, 3–11. Measurements are given in Table 1. ***Coloration*** (Fig. 1). General coloration brownish black to blackish with orange to yellowish and reddish markings on portions of body, whereas the hemelytra are pale greenish with extensive darkened markings. ***Head***: blackish, clypeus reddish on approximately its apical half; a red brownish median longitudinal band, which runs from the space between ocelli to anterior portion, near antennifer tubercles, where it diverges laterally, forming a figure similar to a “T” or a “Y”; area around dorsal portion of eyes with same coloration; mandibular plates, apex of labial segment IV, lateral and ventral portions of collum somewhat paler, red brownish to yellow-brownish, respectively. ***Thorax***: blackish; fore lobe of pronotum with anterolateral angles, discal and lateral tubercles and a straight marking above the latter, and dorsal surface of humeral angles reddish; hind lobe of pronotum with the following orange yellowish markings: a pair of small irregular spots, on anterior portion of submedian carinae, adjacent to transverse sulcus; a somewhat large pair, in which each spot lies between submedian carinae and humeral angle on posterior half, a larger posterior median suboval spot, which ends on posterior margin; a very thin stripe on posterior margin which becomes somewhat larger at the end of submedian carinae on posterior margin and interrupted sublaterally. Rounded reddish to yellow reddish spot on supracoxal lobes, larger on fore lobe and progressively somewhat smaller on middle and hind lobes; a median yellowish marking on posterior margin of mesosternum adjacent to metasternum. Scutellum with posterior process reddish. Legs: blackish to black brownish; trochanters with external portion, mainly adjacent to femora, yellowish; extreme base of femora, adjacent to respective trochanter, somewhat paler; apex of femora reddish on dorsal and lateral surfaces; base of segment II of all tarsi somewhat paler. Hemelytra: greenish or pale green with extensive blackish to brownish markings in a mottled pattern, including larger dark spots on basal, lateral, and apical portions of corium; brownish spots on basal portion of membrane cells and darkened lines and connecting spots over, parallel or between veins of corium. ***Abdomen*.** Connexivum segments with a reddish tint; each segment with a large median subquadrate to subrectangular blackish spot, which is smaller and subtriangular on segment II, and is located on mid portion of basal half on the last segment; these large median black spots reach outer margin of connexivum and its medial suture on ventral portion, but are far from the medial suture in dorsal portion for a distance approximately the same as transverse width of the spot; portion between the black median spot and medial suture brownish; in the last segment, a small and faint dark spot on its median portion, distally; additionally, each connexival segment with a basal thin blackish stripe and distally pale to yellowish; the basal dark stripe variably enlarged on inner portion, except on segment II. Sternites blackish to brownish black with spiracles and area just around them yellowish. Genital segments darkened. ***Vestiture***: body integument covered with numerous adpressed golden, yellowish, or somewhat darkened simple setae (Fig. 3). ***Head*** with short adpressed golden setae, which are absent or very sparse on dorsal blackish portion between eyes. Antennae: segment I with numerous adpressed darkened setae, sparser on ventral side and more numerous on apical margins of dorsal side of the segment; segment II covered with curved somewhat more elongate setae and very numerous, short, thin, whitish setae on anterior and ventral portions; segment III covered with very numerous, short, thin, whitish setae and sparser long, darkened, almost straight setae; segment IV absent. Labium covered by longer, thin, curved setae, which are progressively more numerous towards apical portion of segment III and segment IV; labial segment IV with scattered longer setae too. Neck glabrous. ***Thorax*** and ***abdomen*** covered with short adpressed golden setae, which become somewhat thinner and paler on ventral portions of thorax, sternites and femora; glabrous areas on smooth portions of fore lobe of pronotum, mid and distal portion of clavus of hemelytra, lateral portions of mesosternum and irregular lateral areas of sternites; membrane of hemelytra completely glabrous. Tibiae and tarsi with more numerous, thicker, and darker setae, which become somewhat reddish on apex of tibiae and tarsi, ventrally, where they are a little longer and even more numerous too. Laterobasal small patches of very thin and numerous short yellowish setae on metasternum and sternite II, just below middle and hind coxae. ***Structure*: *Head***: with rugous integument; slightly shorter than pronotum (ratio head/pronotum length: 1:1.08–1.09); longer than larger (ratio head length/width across eyes: 1: 0.67–0.7); anteocular portion length between 3–4 × the postocular region, with respective ratio: 1:0.28–0.30; eyes globose, in lateral view slightly surpassing level of ventral margin but not reaching dorsal outline of head; ratio width of an eye/interocular transverse distance (synthlipsis): 1:1.84–2.4; clypeus larger on posterior half, with anterior margin almost transversely straight. Antennae: ratio of antennal segments (I–III): 1:2.87–3.3:2.5; segment I not surpassing clypeus, somewhat curved and thickened to the apex; segments II and III subcylindrical, the latter thinner than the former; segment IV absent in all specimens. Dorsal area in which there is a median longitudinal brownish to reddish band, with integument more rugous and somewhat elevated, mainly on the divergent anterior branches. Labium straight, reaching stridulatory sulcus at its anterior half, ratio of segments: 1:2.3–2.7:0.7–0.8. ***Thorax***: anterior collar well developed, with integument very finely rugous, subrounded anterolateral angles prominent, compressed dorsoventrally; integument of fore lobe of pronotum almost only rugous on its ridges, on which setae are present; discal and lateral tubercles prominent, rounded; a very shallow crest above lateral tubercles; transverse (interlobar) sulcus large and deep; longitudinal median sulcus linear, extending from anterior margin of fore lobe to approximately basal third of hind lobe of pronotum; hind lobe of pronotum ~ 2.5 × as long as fore lobe, with integument coarsely rugous; submedian carinae shallow, a little larger on basal third; humeral angles prominent somewhat subangular; pleural and sternal integument slightly rugous, completely smooth and shiny on lateral portions of meso-and metasternum; mesosternum with a conical, prominent median protuberance. Scutellum subtriangular with shallow carinae, integument rugous; apex of its process small and rounded. Hemelytra not attaining tip of abdomen by a short distance. Fore trochanter with a basomedial small spine on anterior portion, adjacent to anterior edge of fore coxa. Femora somewhat thickened; fore femora 4.7–4.8 × as long as wide; at apex of all femora, a pair of very small laterodorsal prominences; on ventral submedian distal portion of fore and middle femora, laterally to small shallow glabrous areas, a pair of small prominences variably developed, as small teeth with a terminal seta in the paratypes and sometimes united by a thin shallow ridge. Tibiae straight, thinner; fore tibia thicker at apex, with a mesal distal comb and four to five short spines on distal fifth, ventrally, which may be not easy to distinguish from the very numerous and thicker setae implanted in this portion of the segment; middle tibiae very slightly thicker at apex; spongy fossa very small, with ~ 7–8% (foreleg) to 5–6.5% (middle leg) of respective tibial length. ***Abdomen***: sternites somewhat flattened on median portion; integument finely striated transversely; spiracles small, very close to connexival suture. **Male genitalia** (Figs 4–11): pygophore sub-squared; parameres apices close in resting position. Median process of pygophore weakly sclerotized, subtriangular, pointed to apex (Fig. 6). Parameres symmetrical, curved, with a subapical very small sclerotized pointed and curved tooth; several setae on outer and inner surface of distal two thirds (Figs 4, 5). Phallus with articulatory apparatus moderately short, basal plate arms (bpa) slightly converging towards apex; basal plate bridge (bpb) and median bridge (mb) narrow; pedicel (pd) subretangular (Figs 7, 8). Dorsal phallothecal plate (dpp) somewhat enlarged to the apex, suboval in shape (Fig. 8), with a pair of lateral flap like prominences (flp) at apex; its intermediate portion (ip), between apical half of flap like prominences and main portion of phallothecal plate, much less sclerotized (Figs 7–9); on the ventral portion of the lateral flap like prominence, a moderately elongate process (ep) is present (Figs 8–9); struts (st) subparallel, united at base, somewhat diverging to the apex, in which they are separated (Fig. 8). Endosoma with pair of lateral smooth processes (lp) on approximately median portion (Figs 7, 10) and a subapical median (sp) moderately developed process, which has fine stripes on posterior view (Figs 7, 11).

**Table 1.** Measurements (mm) of male specimens (*n* = 3) of *Panstrongylusnoireaui* sp. nov.

	Holotype	Paratype 1	Paratype 2	Mean
Total length	20.7	19.8	19.6	20.04
Head length	3.4	3.2	3.2	3.27
Anteocular portion	1.8	1.7	1.65	1.72
Postocular portion	0.5	0.5	0.5	0.5
Head width across eyes	2.3	2.4	2.3	2.33
Interocular distance (synthlipsis)	1.2	1.1	1.1	1.17
Right eye: dorsal transverse width	0.6	0.5	0.6	0.57
Right eye: length on dorsal view	1.0	1.0	0.9	0.97
External distance between ocelli	1.3	1.4	1.3	1.33
Antennal segment I	0.7	0.7	0.8	0.73
Antennal segment II	2.3	2.3	2.3	2.3
Antennal segment III (*n* = 1)	1.8	Abs.	Abs.	1.8
Antennal segment IV (*n* = 0)	Abs.	Abs.	Abs.	–
Labium segment II	0.9	0.9	1.0	0.93
Labium segment III	2.4	2.4	2.3	2.37
Labium segment IV	0.7	0.7	0.7	0.7
Pronotum total length	3.7	3.5	3.5	3.57
Pronotum fore lobe length	1.1	1.0	1.0	1.03
Pronotum hind lobe length	2.6	2.5	2.5	2.53
Pronotum maximum width	5.5	5.0	5.0	5.17
Scutellum length	2.2	2.0	1.9	2.03
Fore femur	3.5	3.4	3.5	3.47
Fore femur max. width	0.75	0.7	0.7	0.72
Fore tibia	3.6	3.4	3.4	3.47
Spongy fossa of fore tibia	0.25	0.25	0.3	0.27
Fore tarsus	1.3	1.4	1.5	1.4
Middle femur	3.8	3.5	3.5	3.6
Middle tibia	3.9	3.8	3.7	3.8
Spongy fossa of middle tibia	0.25	0.2	0.2	0.23
Middle tarsus	1.4	1.4	1.4	1.4
Hind femur	5.0	4.7	4.8	4.83
Hind tibia	6.2	5.9	5.7	5.93
Hind tarsus	1.5	1.5	1.5	1.5
Abdomen length	11.2	10.8	11.0	11.0
Abdomen max. width	7.1	6.3	6.0	6.47

**Female.** Fig. 2. Measurements in Table 2. Similar to male. ***Coloration*** (Fig. 2): mandibular plates darkened; because the last segment of connexivum is shorter than in male, the subquadrate median blackish spot is almost centrally located, similarly to the similar same spots of the other segments. Genital segments darkened, with apical portion paler. ***Structure***: ratio head/pronotum length: 1:1.06; anteocular portion length between 3–4 × the postocular region with respective ratio: 1: 0.26–0.30; ratio head length/width across eyes: 1:0.66–0.69; ratio width of an eye/interocular transverse distance (synthlipsis): 1:2.6; antennal segments ratio: 1:3.1–3.4 [segments III–IV absent in all female specimens]; labial segments ratio: 1:2.5–2.9:0.7–0.9; hind lobe of pronotum ~ 2 × as long as fore lobe. Fore femora 4.9–5.1 as long as wide; spongy fossae absent. **Female genitalia**: dorsal view: tergites VII, VIII, IX and X distinctly separated from each other; posterior margins of tergite VII and VIII somewhat concave and slightly curved at median portion, respectively; size of segment X ~ 1/3 that of the preceding segment (IX), both forming a set with subtrapezoidal shape. Ventral view: posterior margin of sternite VII curved backwards on median portion; gonocoxites VIII subtriangular, apical margins rounded; sternites IX barely visible; gonapophysis VIII short, apices rounded. Posterior view: gonocoxites VIII elongate, narrow, slightly wider at median portion; posterior margin of tergite IX well marked, clearly separating it from the following segment (X), these segments combined longer than wide and turned down, perpendicular to the plane of the body.

**Table 2.** Measurements (mm) of female specimens (*n* = 2) of *Panstrongylusnoireaui* sp. nov.

	Paratype female 1	Paratype female 2	Mean
Total length	21.8	21.1	21.45
Head length	3.6	3.5	3.55
Anteocular portion	2.0	1.9	1.95
Postocular portion	0.6	0.5	0.55
Head width across eyes	2.5	2.3	2.4
Interocular distance (synthlipsis)	1.3	1.3	1.3
Right eye: dorsal transverse width	0.5	0.5	0.5
Right eye: length on dorsal view	1.0	1.0	1.0
External distance between ocelli	1.4	1.4	1.4
Antennal segment I	0.8	0.7	0.75
Antennal segment II	2.5	2.4	2.45
Antennal segment III (*n* = 0)	Abs.	Abs.	–
Antennal segment IV (*n* = 0)	Abs.	Abs.	–
Labium segment II	0.9	1.0	0.95
Labium segment III	2.6	2.5	2.55
Labium segment IV	0.8	0.7	0.75
Pronotum total length	3.8	3.7	3.75
Pronotum fore lobe length	1.2	1.2	1.2
Pronotum hind lobe length	2.6	2.5	2.55
Pronotum maximum width	5.7	5.2	5.45
Scutellum length	2.0	1.9	1.95
Fore femur	3.9	3.6	3.75
Fore femur max. width	0.8	0.7	0.75
Fore tibia	3.8	3.6	3.7
Spongy fossa of fore tibia	Abs.	Abs.	–
Fore tarsus	1.5	1.5	1.5
Middle femur	3.9	3.8	3.85
Middle tibia	4.2	4.0	4.1
Spongy fossa of middle tibia	Abs.	Abs.	–
Middle tarsus	1.5	1.5	1.5
Hind femur	5.2	5.2	5.2
Hind tibia	6.5	6.5	6.5
Hind tarsus	1.5	1.6	1.55
Abdomen length	12.2	12.0	12.1
Abdomen max. width	7.1	7.0	7.05

###### Distribution.

Bolivia.

###### Etymology.

The species is named in memory to Dr. François Noireau, a prolific researcher in ecology of Triatominae, who passed away in 2011.

###### Comments.

With exception of the absence of spongy fossa in the female, which is recorded for most species of Triatominae, including *P.rufotuberculatus* (Lent and Wygodzinsky 1979), the subtle differences recorded here between males and females, if attributable to intraspecific or sexual variation, will only be known if (or when) more specimens are examined in the future.

##### ﻿ Panstrongylus rufotuberculatus

Taxon classificationAnimaliaHemipteraReduviidae

(Champion, 1899)

91A85C4D-0A70-57BC-A2BC-47053C039F03

Figs 12–16

###### Material examined.

*Panstrongylusrufotuberculatus* (Champion, 1899). Costa Rica, 1 male, Puntarenas, Est. Queb. Bonita, Res. Biol. Carara, 50 m, L–N 194500, 469850, VI.1992, J. C. Saborio [*leg.*], / *Panstrongylusrufotuberculatus* (Champion), R. Carcavallo det., 1997 / Costa Rica INBIO, CR 1000 872011 [barcode] / 3751; Panama, 1 male, Barro Colorado, C. Z., 18.III.1936 / collected by Gertsch. Lutz, Wood / *Panstrongylusrufotuberculatus* (Champion, 1899), X-946, H. Lent det. / N° 733, HEMIPTERA, Inst. Oswaldo Cruz; Venezuela, 1 male, Cojedes, XI.[19]73, M [?] lrrique / Coleção Rodolfo Carcavallo [Green label] / 3760; 1 male, Estado Falcón, município Colina, Lugar: Puerto Novo, VII.[19]57 / *Panstrongylusrufotuberculatus* (Champion, 1898), det. M. A. Soares / 3015; Ecuador, 1 male, Guayaquil, Sta. [?] Liceia, 08.X... / *P.rufotubercul* [...] / Coleção Rodolfo Carcavallo [Green label] / 3749; Peru, 1 male, Cusco, Convención, 5.VIII.[19]70, Coll. F. carrasco / Vivienda / 3014; Bolivia, La Paz, 2 males, 4 females, Carrasco, 6/93 [VI.1993], *P.rufotuberculatus* Dom. / 3019 (1 male), 3020, 3026 (2 females); 1 female, Tojima, Licoma, 5/94 [V.1994] / 3025 (CTIOC).

**Figures 12–16.**
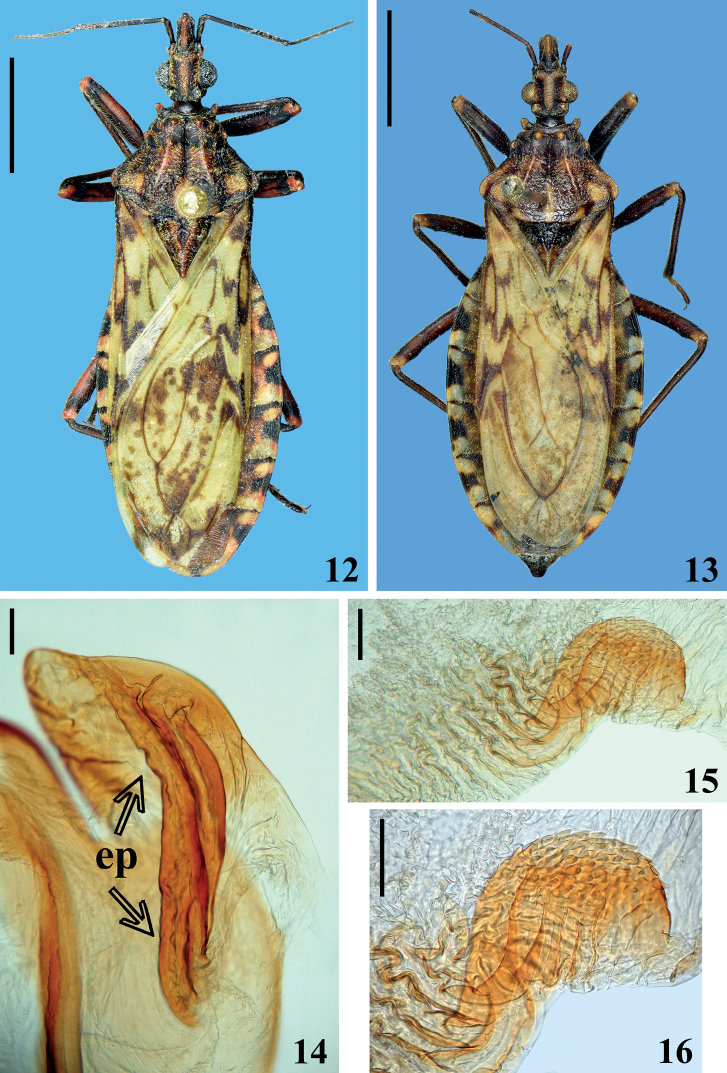
*Panstrongylusrufotuberculatus* (Champion, 1899). **12, 13** habitus dorsal view **12** male from Costa Rica **13** female from Bolivia **14–16** male genitalia **14** latero-apical portion of dorsal phallothecal plate (flap like prominence), ventral view, **ep** elongate process **15, 16** lateral process of endosoma, lateral view **16** detail of the portion with denticulate processes. Scale bars: 0.5 mm (**12, 13**); 0.1 mm (**14–16**).

###### Remarks.

Besides reviewing all previous description and thorough redescriptions of *P.rufotuberculatus* (Champion 1899; Lent and Pifano 1940; Lent and Jurberg 1975; Lent and Wygodzinsky 1979), 13 specimens of this species (eight males and five females from different countries) deposited at CTIOC were examined (e.g., Figs 12, 13). Selected measurements of specimens examined in this work are presented in Tables 3 and 4. Data on morphological variation observed to *P.rufotuberculatus* were summarized together with previous synthesis by Salomón et al. (1999) in Table 5. The female genitalia of *P.noireaui* sp. nov. was compared with the female genitalia of specimens of *P.rufotuberculatus* examined and also with the results recorded by Rodrigues et al. (2018) to the latter species, and no difference was found between them. Male genitalia of four males representing extremes of size (as selected by total length) and from different countries (18 mm, 21 mm, Venezuela; 24.5 mm, Panama; 25 mm, Costa Rica, respectively) were dissected in order to ascertain possible intraspecific variation. Although the general morphology of the male genitalia of *P.rufotuberculatus* seemed similar to its description by Lent and Jurberg (1975), three observations deserve to be recorded. Firstly, the presence of a pair of finely and densely denticulate lateral endosoma processes was confirmed (Figs 15, 16). Secondly, bigger males have more sclerotized structures, including, for example, the phallothecal plates. The most striking, indeed is the (subapical) median process of endosoma (“vesica” *sensu* auths.), which although with similar shape definitively was shown to be increasingly larger how much bigger is the male examined. Thirdly, it was verified that, in contrary to Lent and Jurberg (1975) and Lent and Wygodzinsky (1979) assumptions, there is no second or distal pair of endosoma processes. By comparing the figs 1, 203, and 209 of Lent and Jurberg (1975) with all genitalia dissected in this study, it becomes evident that what was interpreted by them as a second or distal pair of lateral endosoma processes are in fact the pair of lateral flap-like prominences (flp) of the dorsal phallothecal plate (dpp), including a moderately elongate process (ep) which is present on the ventral portion of these lateral flap like prominences (Fig. 14). It is noteworthy that between these flap-like process and the main portion of phallothecal plate there is an intermediate portion which is much less sclerotized and is prone to be easily broken or fractured in the dissecting process. In this latter case, an artifact can be created, and the observer will possibly misinterpret this flap like portion of phallothecal plate as an independent structure, what it is not. On the other hand, the ventral elongate process is clearly connected to the ventral portion of the dorsal phallothecal plate and is not a part of the endosoma, nor a process of it (Fig. 14).

**Table 3.** Selected measurements (mm) of male specimens (*n* = 8) of *P.rufotuberculatus*.

	Maximum	Minimum	Mean	SD
Total length	25.0	18.0	22.7	2.33
Head length (excluding collum)	4.2	3.0	3.7	0.37
Anteocular portion length	2.3	1.6	1.9	0.2
Postocular portion length	0.9	0.5	0.6	0.13
Head width across eyes	2.9	2.2	2.7	0.23
Interocular (synthlipsis)	1.5	1.0	1.28	0.14
Right eye: transverse width	0.8	0.6	0.67	0.08
Antennal segment I	1.1	0.7	0.95	0.13
Antennal segment II (*n* = 7)	4.0	2.5	3.27	0.50
Antennal segment III (*n* = 1)	3.5	3.5	–	–
Antennal segment IV (*n* = 1)	2.5	2.5	–	–
Labium segment II	1.2	0.8	1.1	0.14
Labium segment III	2.7	1.9	2.4	0.27
Labium segment IV	0.9	0.7	0.78	0.09
Pronotum length	5.0	3.5	4.4	0.50
Pronotum maximum width	6.7	4.7	5.9	0.62
Fore femur length	4.9	3.8	4.28	0.4
Fore femur maximum width	1.0	0.7	0.88	0.09
Abdomen maximum width	9.0	5.7	7.73	1.18

**Table 4.** Selected measurements (mm) of female specimens (*n* = 5) of *P.rufotuberculatus*.

	Maximum	Minimum	Mean	SD
Total length	27.5	25.5	26.3	0.74
Head length (excluding collum)	4.2	4.0	4.14	0.08
Anteocular portion length	2.4	2.3	2.34	0.05
Postocular portion length	0.8	0.7	0.74	0.05
Head width across eyes	2.8	2.7	2.76	0.05
Interocular distance (synthlipsis)	1.6	1.5	1.54	0.05
Right eye: transverse width	0.6	0.6	0.6	0.0
Antennal segment I	1.0	0.9	0.92	0.04
Antennal segment II (*n* = 4)	3.5	3.3	3.4	0.08
Antennal segment III (*n* = 0)	–	–	–	–
Antennal segment IV (*n* = 0)	–	–	–	–
Labium segment II	1.2	1.1	1.18	0.04
Labium segment III	2.8	2.6	2.68	0.10
Labium segment IV	0.9	0.7	0.84	0.89
Pronotum length	5.2	4.4	4.82	0.28
Pronotum maximum width	7.0	6.2	6.6	0.3
Fore femur length	4.9	4.5	4.62	0.18
Fore femur maximum width	1.0	0.9	0.94	0.05
Abdomen maximum width	9.5	8.5	9.0	0.5

**Table 5.** Comparisons between specimens of *Panstrongylusrufotuberculatus* studied by Lent and Wygodzinsky (1979), Salomón et al. (1999) and present work. Modified from Salomón et al. (1999).

Character	Lent and Wygodzinsky	Salomón et al.	Present work*
Male length	24–27 mm	23.31 mm	18–25 (22.7) mm
Female length	25–28 mm	23.95 mm	25.5–27.5 (26.3) mm
Pronotum width	6–7 mm	6.06–6.11 mm	F: 6.2–7.0 (6.6) mm M: 4.7–6.7 (5.9) mm
Male abdomen width	8–9 mm	8.04 mm	5.0–9.0 (7.73) mm
Female abdomen width	9–10 mm	8.98 mm	8.5–9.5 (9.0) mm
Dorsal setae	Different shapes	Like Panama example (scalelike)	Not scalelike
Head length: width	1: 0.65–0.80	1: 0.73–0.74	F: 1:0.66–1:0.67 M: 1:0.67–1:0.80
Head: Pronotum length	1: 1.15–1.45	1: 1.14–1.23	F: 1:1.11–1:1.24 M: 1:1.11–1:1.31
Anteocular: postocular length	1: 0.25–0.35	1: 0.21–0.24	F: 1:0.29–1:0.35 M: 1:0.26–1:0.39
Apex of clypeus	Uni or bilobed	Unilobed	Bilobed (3 M) Unilobed (5 F / 5 M)
Eye width: synthlipsis	1: 1.30–1. 85 1:2.3–3.3^α^	1: 1.88–1.94	F: 1:2.50–1:2.67 M: 1:1.62–1:2.67
Antennal first segment	Slightly surpassing apex of clypeus (SC)	Not surpassing apex of clypeus (NSC)	NSC: 5 F / 6 M SC: 2 M
Antennal segments	1: 3.0–3.5:2.2–2.8: 1.9–2.3	1: 2.8–3.1:2.3–2.4: 1.9–2.0	F: 1:3.5–3.7: –:– M: 1:3.2–3.6:2.9–3.2:2.2–2.3
Labium segments	1: 1.9–2.2: 0.6–0.7	1: 2.4: 0.8	F: 1:2.3–2.5:0.6–0.7 M: 1:2.0–2.5:0.6–0.9
Pronotum color	Dark brown to black	Black	Dark brown: 3 F / 4 M Black: 2 F /4 M
Humeral angle	Narrowly rounded (NR) to subangular	Subangular (SA)	NR:4 M SA: 5 F / 4 M
Scutellum posterior process	Apically (AR) or entirely red	Entirely red (ER)	AR: 1 M ER: 5 F / 7 M
Scutellum central carinae	Red or black	Black	Red: 3 M Black: 5 F / 5 M
Scutellum apex	Rounded, suboval or subglobose	Suboval	Rounded: 5 F / 6 M Suboval: 2 M
Fore femora width: length	1: 3.8–4.7	1: 3.8–4.0	F: 1:4.5–5.4 M: 1:4.2–5.2
Connexivum pattern: median spot	Connected or not along outer margin	Not connected	Not connected: 5 F / 6 M Connected: 2M^ß^

* data obtained from 13 specimens (see material examined): 05 females (F), 08 males (M); values between parenthesis: median value. α: specimens from Cuzco, Peru; ß: connected only on anterior portion of outer margin of segments II–V (one male) or III–IV (one male).

## ﻿Discussion

It is noteworthy that *P.rufotuberculatus* has been considered the only species of *Panstrongylus* to have two paired [lateral] endosoma process (Lent and Jurberg 1975; Lent and Wygodzinsky 1979; Jurberg et al. 2001; Papa et al. 2003; Bérenger and Blanchet 2005; Ayala, pers. comm., 2014). However, as recorded here, the second alleged paired endosoma process described by Lent and Jurberg (1975) was in fact a misinterpretation of the flap like prominence of the dorsal phallothecal plate as well as a moderately elongate ventral process of it. A similar structure was observed in *P.noireaui* sp. nov. Therefore, as in all species of *Panstrongylus* the processes of endosoma have shown to possess only a paired lateral process, it seems that this is a constant feature in this genus. Thus, the consideration of two paired endosoma process as an apomorphy of *P.rufotuberculatus* as proposed by Lent and Wygodzinsky (1979) is not sustainable when confronted with the evidence obtained here. On the other hand, two other apomorphies attributed to *P.rufotuberculatus* by these authors, i.e. presence of body scalelike setae and an apically bilobed clypeus, would need to be confirmed in future more extensive works, given they have been shown not only to be variable but commonly absent (Lent and Wygodzinsky 1979; Salomón et al. 1999; present work).

Because the dissection of the male genitalia is usually carried out on only one specimen of each species (Lent and Jurberg 1985), the variability of these structures may remain unrecorded. Among predatory Reduviidae (Gil-Santana et al. 2013) and particularly Triatominae (Lent and Jurberg 1985; Pires et al. 1998) qualitative differences in some phallic structures of male genitalia have been recorded when more than a male specimen of the same species had its genitalia dissected (e.g., Costa et al. 1997). Therefore, despite not being the main objective of this work, four males of *P.rufotuberculatus* with diverse dimensions and from different localities as well as two males of *P.noireaui* sp. nov. (out of three) were dissected in order to perform a preliminary evaluation of a possible male genitalia variability. While the male genitalia structures of *P.noireaui* sp. nov. were shown to be very similar, in *P.rufotuberculatus* the median process of endosoma (“vesica” *sensu* auths.) was shown to be increasingly larger how much bigger was the male examined. It was strikingly diverse from many previous observations (Lent and Jurberg 1985; Pires et al. 1998; Gil-Santana et al. 2013), which have recorded variations in shape or number of phallic structures but not in their size. Costa et al. (1997), although have recorded that the “vesica” of *Triatomabrasiliensis* Neiva, 1911 was variable in size, did not provide any additional information about other characteristics of the males examined by them possibly related to this variable, such as variation of their body size. The variation in size of the median process of endosoma also contradicts the current evidence to insects in general, which suggests that the allometric slopes of genitalia are lower than those of other body parts, giving rise to the “one size fits all” hypothesis (Eberhard et al. 1998; Schulte-Hostedde and Alarie 2006). Therefore, future studies, possibly including morphometry and dissection of more specimens of *P.rufotuberculatus* and other triatomine species, should be carried out to clarify the preliminary observations recorded here.

When considering all the morphological and chromatic variation recorded to *P.rufotuberculatus* (Lent and Wygodzinsky 1979; Salomón et al. 1999; Hiwat 2014; this work) (Table 5), almost all features of *P.noireaui* sp. nov. lie within part of all variation observed to the former species (Table 6). Yet, while measurements of males of *P.noireaui* sp. nov. are comparable to those of smaller males of *P.rufotuberculatus*, females of the new species have been shown to be distinctly smaller (Table 6). However, until more specimens can be examined in order to confirm (or not) possible differences and/or reach a minimal number to allow statistical inference of data, these differences will not be considered to separate these species from each other.

**Table 6.** Comparisons between specimens of *P.rufotuberculatus* (Lent and Wygodzinsky 1979, Salomón et al. 1999, and present work) and *P.noireaui* sp. nov. Modified from Salomón et al. (1999).

Character	*P.rufotuberculatus*	*P.noireaui* sp. nov.*
Male length	18–27 mm	19.6–20.7 (20.04) mm
Female length	23.95–28 mm	21.1–21.8 (21.45) mm
Pronotum width	4.7–7.0 mm	F: 5.2–5.7 (5.45) mm M: 5.0–5.5 (5.17) mm
Male abdomen width	5.0–9.0 mm	6.0–7.1 (6.47) mm
Female abdomen width	8.5–10 mm	7.0–7.1 (7.05) mm
Dorsal setae	Simple or scalelike	Simple (Fig. 3)
Head length: width	1: 0.65–0.80	F: 1:0.66–1:0.69 M: 1:0.67–1:0.75
Head: Pronotum length	1: 1.11–1.45	F: 1:1.06 M: 1:1.08–1:1.09
Anteocular: postocular length	1: 0.21–0.39	F: 1:0.26–1:0.30 M: 1:0.28–1:0.30
Apex of clypeus	Uni or bilobed	Unilobed
Eye width: synthlipsis	1: 1.3–3.3	F: 1:2.6 M: 1:1.84–1:2.4
Antennal first segment	Not or slightly surpassing apex of clypeus	Not surpassing apex of clypeus
Antennal segments	1: 2.8–3.7:2.2–3.2:1.9–2.3	F: 1:3.1–3.4 M: 1:2.87–3.3:2.5
Labium segments	1: 1.9–2.5:0.6–0.9	F: 1:2.5–2.9:0.7–0.9 M: 1:2.3–2.7:0.7–0.8
Pronotum color	Dark brown to black	Black
Humeral angle	Narrowly rounded to subangular	Subangular
Scutellum posterior process	Apically or entirely red	Entirely red
Scutellum central carinae	Red or black	Black
Scutellum apex	Rounded, suboval or subglobose	Rounded
Fore femora width: length	1: 3.8–5.4	F: 1:4.9–5.1 M: 1:4.7–5.0
Connexivum pattern: median spot	Connected or not along outer margin	Not connected

*data obtained on 05 type specimens (see material examined): 02 females (F), 03 males (M); values between parenthesis: median value.

Besides the difference between the ventral process of flap like prominence of phallothecal plate (Figs 9, 14), the smooth lateral endosoma process of *P.noireaui* sp. nov. (Fig. 10) in comparison with the finely and densely denticulate lateral endosoma process of *P.rufotuberculatus* (Figs 15, 16) is an objective and reliable morphological feature to separate these species. It is noteworthy that analogous taxonomic situations occur among other species of Triatomini as follows. *Triatomamaculata* (Erichson, 1848) and *T.pseudomaculata* Corrêa & Spínola, 1964 are extremely similar and cannot be easily distinguished from each other based on external characters alone; only the striking structural differences of the endosomal processes of the phallus [lacking apical teeth, with ribbon-shape sclerotizations in *T.maculata* and delicately striate and denticulate in *T.pseudomaculata*] allow secure identification (Lent and Wygodzinsky 1979). Similarly, *Triatomaarthurneivai* Lent & Martins, 1940 and *T.wygodzinskyi* Lent, 1951 are very similar chromatically and in their external morphology, with minor color differences, which may be subject of slight variation. However, the structure of endosoma processes (with ~ 100 teeth in the first species and ~ 20 in the latter) is sufficient to differentiate these species (Lent and Wygodzinsky 1979).

Thereby, although *P.noireaui* sp. nov. has proved to be morphologically very similar to *P.rufotuberculatus*, the differences observed in male genitalia are analogous to other cases of close morphological species included in Triatomini as referred just above. Therefore, their separation based in differences of the male genitalia, even if considered by themselves alone, would have good grounds in the taxonomy of Triatominae. At the genetic level, *P.noireaui* sp. nov. presents very different chromosomal and molecular characteristics compared to the other *Panstrongylus* species studied so far (Pita et al. 2021) (Table 7). Out of the eight *Panstrongylus* species cytogenetically described, *P.noireaui* sp. nov. is the only one that has a simple sex male mechanism (XY), while the other *Panstrongylus* have multiple sex chromosomes (X_1_X_2_Y or X_1_X_2_X_3_Y) (Panzera et al. 2021). In addition, the chromosomal location of the 45S ribosomal DNA clusters is distinctive character of this new species. In *P.rufotuberculatus*, like all *Panstrongylus* species analyzed hitherto, the rDNA clusters are localized on an autosomal pair, while in *P.noireaui* sp. nov. are localized in both sex chromosomes (X and Y). Both chromosomal markers (sex chromosome system and location of ribosomal clusters) are species-specific characters, being their variation within the same species an exceptional event (Pita et al. 2021, 2022). Moreover, mitochondrial DNA markers showed a remarkable genetic diversity between this new species with all others *Panstrongylus* species, higher than the expected for conspecific populations (Pita et al. 2021). Sequence analyses of cyt b and coI fragments revealed high nucleotide divergence between *P.noireaui* sp. nov. with *P.rufotuberculatus* samples from Colombia, Ecuador, and Mexico, showing Kimura 2-parameter distances higher than 10% (10.7–18.7% for cyt b and 10.6–15.8% for coI) (Pita et al. 2021). Therefore, the morphological evidence presented here is in agreement with previously published genetic data, which reveals that *P.noireaui* sp. nov. represents a new species of *Panstrongylus*, morphological and evolutionarily very close to *P.rufotuberculatus*.

**Table 7.** Comparisons between cytogenetic and molecular characteristics between *P.rufotuberculatus* and *P.noireaui* sp. nov. (data from Pita et al. 2021).

Character	*P.rufotuberculatus*	*P.noireaui* sp. nov.
Diploid chromosome number (2n)	F: 24 chromosomes M: 23 chromosomes	F: 22 chromosomes M: 22 chromosomes
Sex chromosome system	F: X_1_X_1_X_2_X_2_ M: X_1_X_2_Y	F: XX M: XY
Chromosome location of 45S ribosomal DNA clusters	One autosomal pair	Both sex chromosomes (X and Y)
Pairwise genetic distance of cyt b sequences among P.rufotuberculatus* and *P.noireaui* sp. nov.	K2p: 10.7–18.7%	
Pairwise genetic distance of coI sequences among P.rufotuberculatus* and *P.noireaui* sp. nov.	K2p: 10.6–15.8%	

*specimens from different localities

### ﻿Key to the species of *Panstrongylus*, based on Lent and Wygodzinsky (1979), Bérenger and Blanchet (2007), Ayala (2009), and Ayala et al. (2014)

**Table:** 

1	Process of scutellum elongate, subcylindrical narrowly tapering apically	**2**
–	Process of scutellum short, rounded, conical or truncate apically	**12**
2	Specimens almost completely black; small red spot on posterolateral angle of connexivum segments and, in some cases, reddish markings on hind lobe of pronotum	***chinai* (Del Ponte, 1929)**
–	Specimens differently colored	**3**
3	Abdomen light colored ventrally, generally with longitudinal series of black spots at least laterally	**4**
–	Abdomen differently colored, without series of black spots	**7**
4	Femora without markings	**5**
–	Femora with median black annuli or with reddish apex	**6**
5	Integument of postocular lateral portion of head smooth; fore lobe of pronotum without distinct black markings; dorsal connexival segments light colored or with small darkened spots	***lenti* Galvão & Palma, 1968**
–	Integument of postocular lateral portion of head rugous; fore lobe of pronotum with a large mid and smaller lateral darkened markings; connexivum with large dark markings on anterior portion of each segment	***martinezorum* Ayala, 2009**
6	Pronotum with humeral angles flattened; femora light brown with a median black annuli	***mitarakaensis* Bérenger & Blanchet, 2007**
–	Pronotum with humeral angles rounded; femora black with apex reddish	***geniculatus* (Latreille, 1811)**
7	Labial segment III [second visible] as long as or shorter than segment II	***tupynambai* Lent, 1942**
–	Labial segment III longer than segment II	**8**
8	Corium yellow except at base and subapically, contrasting with dark gray membrane; interocular distance (synthlipsis) much less than twice as large as width of an eye in dorsal view; femora with slight subapical protuberances	***howardi* (Neiva, 1911)**
–	Corium as dark as membrane, with base and apex light colored; synthlipsis twice or more than twice as large as width of an eye in dorsal view; fore and mid femora with several conspicuous denticles	**9**
9	Fore lobe of pronotum with distinct discal tubercles	**10**
–	Fore lobe of pronotum with only obsolete or without distinct discal tubercles	**11**
10	Anteocular region of head 2.5 × as long as postocular region; general color brownish black with small light markings	***sherlocki* Jurberg, Carcavallo & Lent, 2001**
–	Anteocular region of head twice as long as postocular region; general color yellowish brown with dark brown markings, particularly on pronotum, corium of hemelytra and connexivum	***lutzi* (Neiva & Pinto, 1923)**
11	Anterolateral processes of pronotum very short, blunt; upper surface of head straight, in lateral view; fore and mid femora with 2–3 denticles; lateral borders of pronotum lobes forming an almost continuous line	***diasi* Pinto & Lent, 1946**
–	Anterolateral processes of pronotum elongate, salient; upper surface of head convex, in lateral view; fore and mid femora with more than three denticles; lateral borders of pronotum lobes forming a distinct angle	***guentheri* Berg, 1879**
12	Mandibular plates (juga, auths.) blunt; connexival segments with central dark spot as well as with narrow transverse dark band anteriorly (Figs 1, 2); body integument with numerous golden adpressed setae, mainly dorsally; hemelytra pale greenish (Figs 1, 2)	**13**
–	Mandibular plates with curved hooklike projection; connexival segments with large anterior dark spot; body integument almost entirely glabrous dorsally; general color of hemelytra not greenish	**14**
13	Lateral paired process of endosoma of male genitalia with fine and numerous teeth apically (Figs 15, 16)	***rufotuberculatus* (Champion, 1899)**
–	Lateral paired process of endosoma of male genitalia smooth apically, without teeth (Fig. 10)	***noireaui* sp. nov.**
14	General color black, with reddish or light reddish brown markings, including four on hind lobe of pronotum; third antennal segment shorter than the second	***megistus* (Burmeister, 1835)**
–	General color yellowish to yellowish brown with dark markings, including one median and two pairs of longitudinal dark markings on hind lobe of pronotum; third antennal segment as long as the second	**15**
15	Scutellum yellowish with a black median longitudinal stripe; fore lobe of pronotum without sublateral tubercles	***humeralis* (Usinger, 1939)**
–	Scutellum black with a yellow median longitudinal stripe; fore lobe of pronotum with sublateral tubercles	***lignarius* (Walker, 1873)**

## Supplementary Material

XML Treatment for Panstrongylus noireaui

XML Treatment for Panstrongylus rufotuberculatus

## References

[B1] Abad-Franch F Paucar CA Carpio CC Cuba Cuba CA Aguilar VHM Miles MA (2001) Biogeography of Triatominae (Hemiptera: Reduviidae) in Ecuador: implications for the design of control strategies. Memórias do Instituto Oswaldo Cruz 96(5): 611–620. 10.1590/S0074-02762001000500004 11500757

[B2] Arias AR Monroy C Guhl F Sosa-Estani S Santos WS Abad-Franch F (2021) Chagas disease control-surveillance in the Americas: The multinational initiatives and the practical impossibility of interrupting vector-borne *Trypanosomacruzi* transmission. Memórias do Instituto Oswaldo Cruz 116: e210130. 10.1590/0074-02760210130 PMC9261920 35830010

[B3] Ayala LJM (2009) Una nueva especie de *Panstrongylus* Berg de Venezuela (Hemiptera: Reduviidae, Triatominae). Entomotrópica 24: 105–109.

[B4] Ayala LJM Mattei R Mattei R (2014) Descripción de la hembra de *Panstrongylusmartinezorum* Ayala, 2009 (Hemiptera, Reduviidae, Triatominae) con comentarios sobre la distribución geográfica de la especie en el Estado Amazonas, Venezuela. Boletín de la SEA 54: 383–389. http://sea-entomologia.org/PDF/Boletin54/383389BSEA54HembraPmartinezorumVenezuela.pdf

[B5] Bérenger J-M Blanchet D (2007) A new species of the genus *Panstrongylus* from French Guiana (Heteroptera; Reduviidae; Triatominae). Memórias do Instituto Oswaldo Cruz 102(6): 733–736. 10.1590/S0074-02762007000600012 17924003

[B6] Bérenger J-M Pluot-Sigwalt D Pagès F Blanchet D Aznar C (2009) The Triatominae species of French Guiana (Heteroptera: Reduviidae). Memórias do Instituto Oswaldo Cruz 104(8): 1111–1116. 10.1590/S0074-02762009000800007 20140371

[B7] Berg C (1879) Hemiptera Argentina enumerativ speciesque novas descripsit. Pauli E. Coni, Hamburg, 316 pp. 10.5962/bhl.title.36493

[B8] Champion GC (1899) InsectaRhynchota. Hemiptera-Heteroptera, Fam. Reduviidæ, Subfam. Acanthaspidinæ. Vol II. In: Godman FD Salvin O (Eds) Biologia Centrali Americana.Taylor and Francis, London, 190–211. [pls. XI–XII] [xvi + 416 pp, 22 pls]

[B9] Costa J Barth OM Marchon-Silva V Almeida CE Freitas-Sibajev MGR Panzera F (1997) Morphological Studies on the *Triatomabrasiliensis* Neiva, 1911 (Hemiptera, Reduviidae, Triatominae) Genital Structures and Eggs of Different Chromatic Forms. Memórias do Instituto Oswaldo Cruz 92(4): 493–498. 10.1590/S0074-02761997000400009

[B10] Costa J Dale C Galvão C Almeida CE Dujardin JP (2021) Do the new triatomine species pose new challenges or strategies for monitoring Chagas disease? An overview from 1979–2021. Memórias do Instituto Oswaldo Cruz 116: e210015. 10.1590/0074-02760210015 PMC8186471 34076075

[B11] Dale C Justi AS Galvão C (2021) *Belminussantosmalletae* (Hemiptera: Heteroptera: Reduviidae): new Species from Panama, with an updated key for *Belminus* Stål, 1859 species. Insects 12(8): e686. 10.3390/insects12080686 PMC8396495 34442252

[B12] Eberhard WG Huber BA Rodriguez RL Briceño RD Salas I Rodriguez V (1998) One size fits all? Relationships between the size and degree of variation in genitalia and other body parts in twenty species of insects and spiders. Evolution; International Journal of Organic Evolution 52(2): 415–431. 10.1111/j.1558-5646.1998.tb01642.x 28568329

[B13] Forero D Weirauch C (2012) Comparative genitalic morphology in the New World resin bugs Apiomerini (Hemiptera, Heteroptera, Reduviidae, Harpactorinae). Deutsche Zeitschrift Entomologische 59: 5–41.

[B14] Galvão C (2021) Taxonomy. In: Guarneri A Lorenzo M (Eds) Triatominae – The Biology of Chagas Disease Vectores, Entomology in Focus 5.Springer, Cham, 15–38. 10.1007/978-3-030-64548-9_2

[B15] Galvão C Carcavallo RU Rocha DS Jurberg J (2003) A checklist of the current valid species of the subfamily Triatominae Jeannel, 1919 (Hemiptera, Reduviidae) and their geographical distribution, with nomenclatural and taxonomic notes. Zootaxa 202(1): 1–36. 10.11646/zootaxa.202.1.1

[B16] Gil-Santana HR Davranoglou L-R Neves JA (2013) A new synonym of *Graptocleptesbicolor* (Burmeister), with taxonomical notes (Hemiptera: Heteroptera: Reduviidae: Harpactorini). Zootaxa 3700: 348–360. 10.11646/zootaxa.3700.3.2 26106731

[B17] Gorla D Noireau F (2010) Geographic distribution of Triatominae vectors in America. In: Telleria J Tibayrenc M (Eds) American Trypanosomiasis – Chagas disease, one hundred years of research.Elsevier, London, Burlington, 209–231. 10.1016/B978-0-12-384876-5.00009-5

[B18] Hiwat H (2014) Triatominae species of Suriname (Heteroptera: Reduviidae) and their role as vectores of Chagas disease. Memórias do Instituto Oswaldo Cruz 109(4): 452–458. 10.1590/0074-0276130408 25004146 PMC4155847

[B19] Hypša V Tietz D Zrzavý J Rego RO Galvão C Jurberg J (2002) Phylogeny and Biogeography of Triatominae (Hemiptera, Reduviidae): A molecular evidence of New World origin of the Asiatic clade. Molecular Phylogenetics and Evolution 23(3): 447–457. 10.1016/S1055-7903(02)00023-4 12099798

[B20] Jurberg J Carcavallo RU Lent H (2001) *Panstrongylussherlocki* sp. n. do estado da Bahia (Hemiptera, Reduviidae, Triatominae). Entomología y Vectores 8: 261–274.

[B21] Justi SA Russo CM Mallet JRS Obara MT Galvão C (2014) Molecular phylogeny of Triatomini (Hemiptera: Reduviidae: Triatominae). Parasites & Vectors 7(1): e149. 10.1186/1756-3305-7-149 PMC4021723 24685273

[B22] Kieran TJ Gordon ERL Zaldívar-Riverón A Ibarra-Cerdeña C Glenn TC Weirauch C (2021) Ultraconserved elements reconstruct the evolution of Chagas disease-vectoring kissing bugs (Reduviidae: Triatominae). Systematic Entomology 46(3): 725–740. 10.1111/syen.12485

[B23] Kirkaldy GW (1904) Bibliographical and nomenclatorial notes on the Hemiptera.-No. 3. The Entomologist 37: 279–283. 10.5962/bhl.part.2885

[B24] Lent H Jurberg J (1975) O gênero *Panstrongylus* Berg, 1879, com um estudo sobre a genitália externa das espécies (Hemiptera, Reduviidae, Triatominae). Revista Brasileira de Biologia 35: 379–438.

[B25] Lent H Jurberg J (1985) Sobre a variação intra-específica em *Triatomadimidiata* (Latreille) e *Triatomainfestans* (Klug) (Hemiptera, Reduviidae). Memórias do Instituto Oswaldo Cruz 80(3): 285–299. 10.1590/S0074-02761985000300004

[B26] Lent H Pifano F (1940) Sobre a identidade dos generos *Panstrongylus* Berg, 1879 e *Mestor* Kirkaldy, 1904. Redescrição de *Panstrongylusrufotuberculatus* encontrado, na Venezuela, naturalmente infestado pelo *Schizotrypanumcruzi*. Revista de Entomologia 11(3): 629–639.

[B27] Lent H Wygodzinsky P (1979) Revision of the Triatominae (Hemiptera: Reduviidae) and their significance as vectors of Chagas’ disease. Bulletin of the American Museum of Natural History 163: 123–520. http://hdl.handle.net/2246/1282

[B28] Marcilla A Bargues MD Abad-Franch F Panzera F Carcavallo CU Noireau F Galvão C Jurberg J Miles MA Dujardin JP Mas-Coma S (2002) Nuclear rDNA ITS-2 sequences reveal polyphyly of *Panstrongylus* species (Hemiptera: Reduviidae: Triatominae), vectors of *Trypanosomacruzi.* Infection, Genetics and Evolution 1(3): 225–235. 10.1016/S1567-1348(02)00029-1 12798019

[B29] Marín E Santillán R Cuba C Jurberg J Galvão C (2007) Hallazgo de *Panstrongylusrufotuberculatus* (Champion, 1899) (Hemiptera, Reduviidae, Triatominae) em ambiente domiciliario en la Región Piura, Perú. Intra-domiciliary capture of *Panstrongylusrufotuberculatus* (Champion, 1899) (Hemiptera, Reduviidae, Triatominae) in Piura, Peru. Cadernos de Saude Publica 23(9): 2235–2238. 10.1590/S0102-311X2007000900031 17700958

[B30] Monteiro FA Weirauch C Felix M Lazoski C Abad-Franch F (2018) Evolution, systematics, and biogeography of the Triatominae, Vectors of Chagas Disease. Advances in Parasitology 99: 265–344. 10.1016/bs.apar.2017.12.002 29530308

[B31] Paiva VF Belintani T Oliveira J Galvão C Rosa JA (2022) A review of the taxonomy and biology of Triatominae subspecies (Hemiptera: Reduviidae). Parasitology Research 121(2): 499–512. 10.1007/s00436-021-07414-2 34984541

[B32] Panzera F Pita S Lorite P (2021) Chromosome structure and evolution of Triatominae: a review. In: Guarneri A Lorenzo M (Eds) Triatominae – The Biology of Chagas Disease Vectores, Entomology in Focus 5.Springer, Cham, 65–99. 10.1007/978-3-030-64548-9_2

[B33] Papa AR Barata JMS Obara MT Ceretti Jr W Jurberg J (2003) Descrição da genitália externa do alótipo macho *Panstrongyluslenti* Galvão & Palma, 1968 (Hemiptera, Reduviidae, Triatominae). Entomología y Vectores 10: 345–352.

[B34] Patterson JS Barbosa SE Dora Feliciangeli M (2009) On the genus *Panstrongylus* Berg 1879: Evolution, ecology and epidemiological significance. Acta Tropica 110(2–3): 187–199. 10.1016/j.actatropica.2008.09.008 18929527

[B35] Pinto C (1931) Valor do rostro e antenas da caracterização dos generos de Triatomideos. Hemiptera. Reduvidioidea. Boletín Biológico 19: 45–136.

[B36] Pires HHR Barbosa SE Margonari C Jurberg J Diotaiuti L (1998) Variations of the external male genitalia in three populations of *Triatomainfestans* Klug, 1834. Memórias do Instituto Oswaldo Cruz 93(4): 479–483. 10.1590/S0074-02761998000400011 9711336

[B37] Pita S Gómez-Palacio A Lorite P Dujardin JP Chavez T Villacís AG Galvão C Panzera Y Calleros L Pereyra-Mello S Burgueño-Rodríguez G Panzera F (2021) Multidisciplinary approach detects speciation within the kissing bug *Panstrongylusrufotuberculatus* populations (Hemiptera, Heteroptera, Reduviidae). Memórias do Instituto Oswaldo Cruz 116: e210259. 10.1590/0074-02760210259 PMC8815762 35137904

[B38] Pita S Lorite P Cuadrado A Panzera Y de Oliveira J Alevi KCCC Rosa JA Freitas SPC Gómez-Palacio A Solari A Monroy C Dorn PL Cabrera-Bravo M Panzera F (2022) High chromosomal mobility of rDNA clusters in holocentric chromosomes of Triatominae, vectors of Chagas disease (Hemiptera-Reduviidae). Medical and Veterinary Entomology 36(1): 66–80. 10.1111/mve.12552 34730244

[B39] Rédei D Tsai J-F (2011) The assassin bug subfamilies Centrocnemidinae and Holoptilinae in Taiwan (Hemiptera: Heteroptera: Reduviidae). Acta Entomologica Musei Nationalis Pragae 51: 411–442.

[B40] Rodrigues JMS Rosa JA Moreira FFF Galvão C (2018) Morphology of the terminal abdominal segments in females of Triatominae (Insecta: Hemiptera: Reduviidae). Acta Tropica 185: 86–97. 10.1016/j.actatropica.2018.04.021 29684355

[B41] Salomón OD Ripoll CM Rivetti E Carcavallo RU (1999) Presence of *Panstrongylusrufotuberculatus* (Champion, 1899) (Hemiptera: Reduviidae; Triatominae) in Argentina. Memórias do Instituto Oswaldo Cruz 94(3): 285–288. 10.1590/S0074-02761999000300002 10348976

[B42] Schofield CJ Galvão C (2009) Classification, evolution, and species groups within Triatominae. Acta Tropica 110(2–3): 88–100. 10.1016/j.actatropica.2009.01.010 19385053

[B43] Schuh RT Weirauch C (2020) True bugs of the world (Hemiptera: Heteroptera). Classification and natural history. 2^nd^ edn. Siri Scientific Press, Manchester, 767 pp. [32 pls]

[B44] Schuh RT Weirauch C Wheeler WC (2009) Phylogenetic relationships within the Cimicomorpha (Hemiptera: Heteroptera): a total-evidence analysis. Systematic Entomology 34(1): 15–48. 10.1111/j.1365-3113.2008.00436.x

[B45] Schulte-Hostedde A Alarie Y (2006) Morphological patterns of sexual selection in the diving beetle *Graphoderusliberus*. Evolutionary Ecology Research 8: 891–901.

[B46] Usinger RL (1939) Descriptions of new Triatominae with a key to genera (Hemiptera, Reduviidae). University of California Publications in Entomology 7: 33–56.

[B47] Usinger RL (1944) The Triatominae of North and Central America and the West Indies and their Public Health significance. Public Health Bulletin 288: 1–83.

[B48] Weirauch C (2008) From four- to three- segmented labium in Reduviidae (Hemiptera: Heteroptera). Acta Entomologica Musei Nationalis Pragae 48: 331–344.

[B49] Wolff M Castillo D Uribe J Arboleda JJ (2001) Tripanosomiasis americana: Determinación de riesgo epidemiológico de transmisión en el município de Amalfi, Antioquia. Iatreia 14: 111–121.

